# Copro-molecular diagnosis of the Toxoplasmatinae subfamily in dog and cat populations in northern Iran

**DOI:** 10.4178/epih.e2020074

**Published:** 2020-12-04

**Authors:** Leila Izadi, Shahabeddin Sarvi, Seyed Abdollah Hosseini, Afsaneh Amouei, Mehdi Sharif, Mohammad Taghi Rahimi, Tooran Nayeri, Ahmad Daryani

**Affiliations:** 1 Toxoplasmosis Research Center, Mazandaran University of Medical Sciences, Sari, Iran; 2 Department of Parasitology and Mycology, School of Medicine, Mazandaran University of Medical Science, Sari, Iran; 3 Student Research Committee, Mazandaran University of Medical Science, Sari, Iran; 4 Department of Parasitology, School of Medicine, Sari Branch, Islamic Azad University, Sari, Iran; 5 School of Medicine, Shahroud University of Medical Sciences, Shahroud, Iran

**Keywords:** Toxoplasmatinae, Dogs, Cats, Feces, Iran

## Abstract

**OBJECTIVES:**

The oocysts of the Toxoplasmatinae subfamily (*Neospora caninum*, *Hammondia hammondi* and *H. heydorni*, and *Besnoitia besnoiti*) are morphologically similar to *Toxoplasma gondii*, and indistinguishable from each other. This study investigated the prevalence of the Toxoplasmatinae subfamily in dog and cat fecal samples using a nested polymerase chain reaction method.

**METHODS:**

Overall, 200 fecal samples from domestic dogs (n=120) and cats (n=80) were collected from 15 farms in northern Iran. The samples were homogenized in 2.5% potassium dichromate solution and subsequently concentrated with sucrose solution. DNA was extracted from samples using a genomic DNA kit. Specific primers and the 18S rDNA gene were used to screen and detect all Toxoplasmatinae oocysts.

**RESULTS:**

Overall, 2.5% (3 of 120) and 22.5% (18 of 80) of the fecal samples collected from dogs and cats were infected with Toxoplasmatinae. In dogs, 2 samples were positive for *N. caninum* and 1 sample was positive for *T. gondii*. In cats, all 18 positive samples belonged to *T. gondii*. No contamination with *H. heydorni* was observed in dog fecal samples or *H. hammondi* and *B. besnoiti* in cat fecal samples. A phylogenetic analysis revealed that the *T. gondii* (cat) and *N. caninum* (dog) found had similarities with parasites reported from other regions of the world.

**CONCLUSIONS:**

This is the first study to provide data on the epidemiology of Toxoplasmatinae oocysts in Iran. The findings suggest that public-health monitoring for the effective control of feces from cats and dogs and improved pet hygiene habits are needed.

## INTRODUCTION

Tissue-cyst-forming apicomplexan parasites, such as *Toxoplasma gondii*, *Neospora caninum*, Hammondia spp. (*H. hammondi* and *H. heydorni*), and *Besnoitia besnoiti* belong to the Toxoplasmatinae subfamily, which is associated with a variety of diseases in humans and other animals [1].

*T. gondii* is an obligate intracellular protozoan that causes the infectious disease toxoplasmosis. It has 2 distinct life cycles. The sexual cycle occurs only in cats, the definitive host. The asexual cycle occurs in all warm-blooded animals (mammals and birds) and humans. This parasite is pathogenic in human and animals [2]. It is often asymptomatic, but it can lead to severe complications in infants and persons with acquired immune deficiency, especially acquired immune deficiency syndrome (AIDS); its symptoms can include microcephaly, hydrocephalus, and chorioretinitis in infants, as well as repeated attacks of encephalitis in immunodeficient individuals, which may even be fatal. Clinical toxoplasmosis in animals is seen most often in sheep and goats when infected during pregnancy and manifests as abortions, stillbirths, and mummification [3].

*N. caninum* is an obligate intracellular coccidian parasite that causes spontaneous abortion in infected livestock and paralysis in dogs [4,5]. Dogs are currently believed to be the definitive host of *N. caninum*. Intermediate hosts such as cattle can be infected by neosporosis via ingestion of oocysts from the feces of dogs (horizontal transmission) or from the dam to the fetus (vertical transmission). Despite serological evidence of human exposure in immunocompromised individuals, this unicellular organism is not considered to be a zoonotic parasite [6,7].

*H. hammondi* and *H. heydorni* are cyst-forming coccidian protozoans, with very low prevalence rates. Cats are the definitive hosts of *H. hammondi* and dogs, foxes, and coyotes are considered to be the definitive hosts of *H. heydorni*. Unlike *T. gondii* and *N. caninum*, *H. hammondi* and *H. heydorni* are not known to lead to clinical signs in either their definitive or intermediate hosts [8].

*B. besnoiti* is the etiological agent of bovine besnoitiosis and has a significant economic impact on the cattle industry. Currently, knowledge of the definitive host for *B. besnoiti* is limited. However, it seems that cats are the definitive hosts for several *Besnoitia* species that infect wildlife, and cattle represent an important intermediate host for this protozoan [9].

All oocysts of the Toxoplasmatinae subfamily are similar in size (9-14 μm) and morphologically indistinguishable from each other [10,11]. Since their oocysts are physically and morphologically similar to *T. gondii*, and cannot be distinguished from each other [12], a diagnosis based on light microscopy of oocysts in feces is not a method of choice for species identification of these important coccidia. Instead, these oocysts can only be distinguished via molecular methods. Diagnostic molecular techniques, such as polymerase chain reaction (PCR), are a highly sensitive and specific alternative to morphological methods [12-14]. Given the importance of molecular methods, identification of these parasites is crucial for precise control of these zoonotic diseases and making proper decisions regarding economic losses. The aim of this study was to investigate the infection rates of the Toxoplasmatinae subfamily in fecal samples of dogs and cats as reservoir hosts, using nested PCR followed by sequencing and a phylogenetic assay, to demonstrate the circulation of these parasites in northern Iran.

## MATERIALS AND METHODS

### Sample collection

Overall, 200 fecal samples (about 20 g) from domestic dogs (n=120) and cats (n=80) were collected between April 2015 and March 2016 from 15 farms (cattle, sheep, goat) in different region of northern Iran. The fecal samples were transferred to the parasitology laboratory of Mazandaran University of Medical Sciences.

### Purification and sporulation of oocysts

To purify the oocysts, the fecal material was sieved via a strainer (60 meshes) using tap water. Then, for sporulation, fecal samples were mixed with 2.5% potassium dichromate (K_2_Cr_2_O_7_) and incubated at room temperature for 2 weeks.

### Concentration of oocysts

To concentrate the oocysts, they were first washed 3 times with tap water by centrifugation (300× g) for 3 minutes to remove the K_2_Cr_2_O_7_. Then, 1 g of the fecal sample was transferred into a 10-mL tube mixed with 9 mL of 1 M sucrose. The samples were centrifuged at 800× g for 5 minutes. The middle layer was removed and placed in a fresh micro-tube, and after centrifuging at 800×g for 8 minutes, the supernatant was removed and the resulting pellet washed twice with distilled water by centrifuging at 800× g for 5 minutes. The supernatant was decanted and the pellet was resuspended in distilled water at the final volume and stored at -20°C before processing.

### DNA extraction and molecular assay

DNA was extracted from all of the dog and cat fecal samples using the a genomic DNA kit (Dena Zist, Mashhad, Iran) according to the manufacturer’s instructions. To screen and identify the Toxoplasmatinae subfamily, nested PCR was performed to amplify fragments of the 18S rDNA gene with external and internal primers. The external primers were Tg18s48R (5′-CCATGCATGTCTAAGTATAAGC-3′) and Tg18s359R (5′-GTTACCCGTCACTGCCAC-3′), which amplified a 312-bp fragment. The internal primers were Tg18s58F (5′-CTAAGTATAAGCTTTTATACGGC-3′) and Tg18s348R (5′-TGCCACGGTAGTCCAATAC-3′), which amplify a 291-bp region of the 18S rDNA gene [15]. Each amplification reaction was set for a total volume of 25 μL, containing 12.5 μL of 1× PCR mix (Taq PCR Master Mix Kit, Ampliqon, Odense, Denmark), 0.1 mM of each of the external and internal primers and 1.0 μL of template DNA. The final volume (25 µL) was reached by adding nuclease-free water. The PCR conditions in both stages were as follows: initial denaturation for 5 minutes at 94°C; 30 cycles of denaturing for 30 seconds at 94°C, annealing for 30 seconds at 60°C, extension for 30 seconds at 72°C; and final extension for 7 minutes at 72°C.

In the next step, samples that were positive for Toxoplasmatinae subfamily DNA were subsequently subjected to conventional PCR using specific primers for each parasite. For dog feces samples, specific primers for *T. gondii*, *N. caninum*, and *H. heydorni* were used. For cat feces samples, specific primers for *T. gondii*, *N. caninum*, *H. hammondi*, and *B. besnoiti* were used. Each PCR reaction was carried out in a volume of 25 µL, containing 12.5 μL of 1× PCR mix (Taq PCR Master Mix Kit, Ampliqon), 0.1 mM of each of the specific primers, and 70 ng of template DNA. PCR amplification was performed with a primary denaturing step at 94°C for 5 minutes, 30 cycles of denaturing at 94°C for 30 seconds, a specific annealing temperature for each parasite, and extension at 72°C for 90 seconds. After this process, final extension was performed at 72°C for 10 minutes (Table 1). The PCR products were confirmed by visualization on a 1.5% agarose gel with an ultraviolet transilluminator after staining with SYBR Safe (which dyed the PC products green).

### Sequencing and phylogenetic analysis

The preparing of sequences was performed using Chromas version 2.33 (http://www.technelysium.com.au/chromas.html). The Multalin program was applied to compare and align the nucleotide sequences with each other. Phylogenetic analysis was performed by constructing a phylogenetic tree using the Neighbor-Joining method in MEGA version 4.0 (https://www.megasoftware.net/mega4/). The bootstrapping method with 1,000 replicates was used to assess the reliability of the phylogenetic tree.

### Ethics statement

The research presented in this article was approved (No. 1716) by the Deputy of Research, Mazandaran University of Medical Sciences, Sari, Iran.

## RESULTS

**Figure 1.**
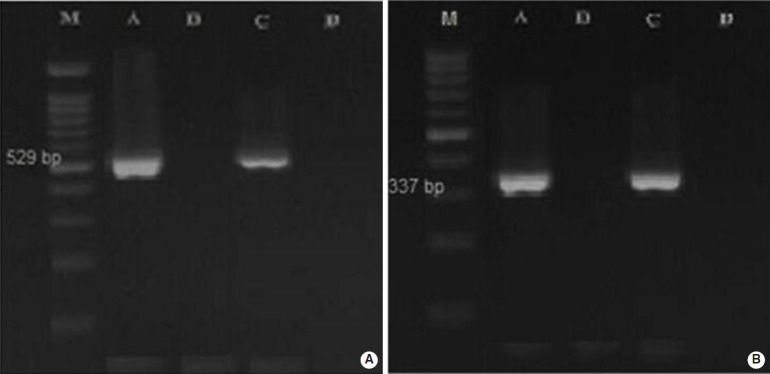
Polymerase chain reaction amplification products of respective (A) *Toxoplasma gondii* and (B) *Neospora caninum* were subjected to 1.5% agarose gel electrophoresis. M: 100-bp DNA ladder, A: positive control, B: negative control, C: positive sample, D: negative sample.

Overall, 2.5% (3 of 120) and 22.5% (18 of 80) of the fecal samples collected from dogs and cats living on farms in northern Iran were infected with Toxoplasmatinae DNA, respectively, based on nested PCR using the 18S rDNA gene. In the dog fecal samples, the presence of *N. caninum* and *T. gondii* DNA was confirmed by the observation a 337-bp band in 2 samples (1.7%) and a 529-bp band in 1 sample (0.8%). However, no contamination with *H. heydorni* was observed. In cat fecal samples, the presence of *T. gondii* DNA was confirmed by observation of a 529-bp band in 18 samples (22.5%) (Figure 1). However, no contamination with *H. hammondi* or *B. besnoiti* was observed (Table 2).

Thirteen of the 21 samples of positive PCR products were sequenced by Sanger sequencing, and it was found that 2 samples of *N. caninum* from the dog fecal samples were registered in the National Center for Biotechnology Information gene bank with accession numbers MN658869 and MN658870. The similarity of *T. gondii* isolated from the dog fecal samples (MN595275) in comparison with the available genes in the gene data bank was 99.2-99.6%. In addition, 10 sequences of *T. gondii* isolated from cat fecal samples were deposited in GenBank under the accession numbers MN595276-MN595285.

**Figure 2.**
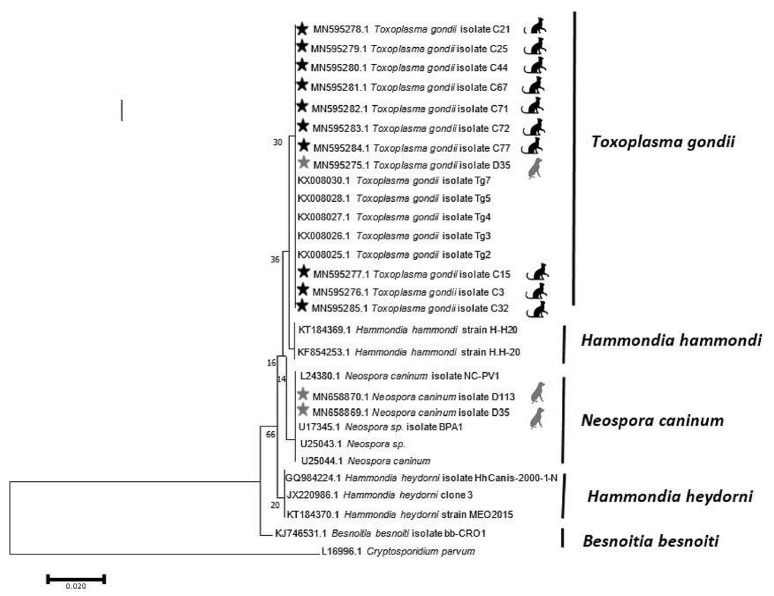
Phylogenetic relationship of *T. gondii* and *N. caninum* isolates obtained in this study and reference sequences retrieved from GenBank, using the maximum likelihood method based on 18S rDNA gene with cryptosporidium parvum as the out-group.

The findings of the PCR assay closely conformed to those of the Basic Local Alignment Search Tool results and phylogenetic tree. An analysis of the phylogenetic tree revealed that the isolates of *T. gondii* found in the dog and cat fecal samples in Mazandaran Province were similar to *T. gondii* isolates from China (accession number: KX008030; host: sheep), Brazil (accession number: KX008028; host: cat), Canada (accession number: KX008027; host: cougar), and France (accession number: KX008026; host: *Homo sapiens*). Moreover, the *N. caninum* observed in dog stool was similar to *N. caninum* isolates reported from the USA (accession number: U17345.1; host: cattle and accession number: U25044.1; host: *Canis familiaris*) (Figure 2).

## DISCUSSION

Due to the similarity of Toxoplasmatinae oocysts, microscopic methods cannot distinguish between their oocysts. To solve this problem, molecular methods are recommended. However, *T. gondii* and *N. caninum* seropositivity in dogs only indicates contact with the protozoan and does not automatically demonstrate oocyst shedding, PCR has been recommended in recent years for better detection of *T. gondii* and *N. caninum* in dogs [20-22].

PCR is a sensitive and specific assay that has been used to identify neosporosis in both definitive and intermediate hosts [13]. Molecular diagnostic techniques such as PCR are the best choice for detecting *H. heydorni* from an epidemiological standpoint, and are important for distinguishing this species from other tissue-cyst-forming apicomplexan parasites [23].

Recent molecular studies based on internal transcribed spacer 1 (ITS1) sequences demonstrated that this gene marker can be used to identify the DNA of apicomplexans that are closely related to *T. gondii* [13]. It has been shown that *N. caninum* genomic DNA could be distinguished from *H. heydorni* using the Np6/N21 primer set. The results of PCR assays using these primers demonstrated that *H. heydorni* DNA was not amplified, and did not interfere with the amplification of *N. caninum* DNA in mixed samples. Slapeta and collegues [13,24] and Dubey et al. [5] designed JS4-JS5 primers that are able to specifically amplify the 3’ end of the small subunit rRNA gene and ITS1 of *H. heydorni*. In this study, we used ITS1 of 18S rDNA as a marker to detect the DNA of apicomplexans that are closely related to *T. gondii* in cat and dog fecal samples. Our results from dog fecal samples showed that 2.5% (3 of 120) of fecal samples were infected with Toxoplasmatinae DNA. Subsequently, we used the special primers (SJ4/SJ5 and N21+/N6+) to distinguish *N. caninum* from *H. heydorni*.

Our molecular survey using N21+/N6+ primers showed that 1.7% of dog feces samples were infected with *N. caninum*. The result is similar to that of a study carried out in Australia by King et al. [18], which used molecular methods and found oocysts of *N. caninum* in 2 out of 132 (1.5%) dog fecal samples. In Iran, 2 studies have been conducted on *N. caninum* oocysts in dog fecal samples. Razmi [25] in the city of Mashhad, reported a prevalence of *N. caninum* of 1.5% (2 of 174 samples) using PCR. In another study carried out in western Iran by Ghafarifar et al. [26], 9 positive samples of *N. caninum* were detected from 428 fecal specimens (2.1%). The prevalence of *N. caninum* seems to be affected by favorable geographical conditions, the density of the dog population, and the presence of intermediate hosts in the surrounding environment. Dogs can shed oocysts in the environment of farms. *N. caninum* is considered to be one of the most important infectious agents responsible for abortion in cattle. Exposure of cattle to *N. caninum* oocysts causes economic losses due to reproductive failure associated with abortion. Therefore, farm owners should be aware of the potential risks posed by the presence of stray dogs around the farm.

In the present study, *H. heydorni* was not detected in the fecal samples of domestic dogs. Our results were not unexpected because to our knowledge, the prevalence rate of *H. heydorni* in dogs in various countries is very low: 0.05% in Germany [4], 0.2% in the Czech Republic [13], and 0.8% in China [27]. However, Li et al. [28] in a rural region of China, reported a seemingly unusually high prevalence of *N. caninum* in dog feces (17.5%). In our study, *T. gondii* DNA was surprisingly observed in a feces sample from 1 dog. This result shows that dog feces could pose a risk of toxoplasmosis to other species, including humans; and dogs may serve as mechanical vectors for this parasite [29]. Since dogs are not the definitive host for *T. gondii*, and its sexual cycle has not been observed, they are not able to shed oocysts. Therefore, in the context of this cross-sectional study, it seems that the dog had ingested *T. gondii* oocysts by coprophagia [4]. Our results of cat fecal samples showed that 22.5% (18/80) of the samples were infected with Toxoplasmatinae DNA. We subsequently used special primers (TOX4/TOX5, Hham3F/Hham3R, ITS-1F/ITS-1R) to distinguish *T. gondii* from *H. hammondi* and *B. besnoiti*. In the cat fecal samples, the presence of *T. gondii* DNA was confirmed by the observation of a 529-bp band in 18 samples (22.5%), while no contamination with *H. hammondi* or *B. besnoiti* was observed.

The prevalence of *T. gondii* in the present study in cats is very similar to that reported by Mancianti et al. [30], who reported a prevalence of 16% (8 positive out of 50 cat fecal samples) in a rural area of Italy. Overall, the prevalence of *T. gondii* in cat fecal samples in most studies ranges from 0% to 5%. In a study by Berger-Schoch et al. [31] in Switzerland, the prevalence of this parasite was found to be 0.4% using molecular PCR. Jokelainen et al. [32] also reported a prevalence of 1.5% in Finland using microscopic and molecular methods. According to a literature review, the highest prevalence of this parasite in cat fecal samples was reported by Dubey et al. [33] in Ethiopia, with a prevalence of 19.4%. According to the above findings, the observed prevalence of *T. gondii* in cat fecal samples in our study in Mazandaran Province is very high. It seem that *T. gondii* infections in cats could be caused by the complicated environment of farms, accidental ingestion of undercooked food, and the presence of other intermediate hosts such as sheep and goats [3].

It seems that the performance of high sensitivity molecular tests and the use of the RE gene marker, which is repeated about 200 times to 300 times throughout the *T. gondii* genome [34], was very effective for identifying the high prevalence of *T. gondii* in this study. Furthermore, other studies of this important zoonotic parasite in different human and animal groups in Mazandaran Province have confirmed its high prevalence. For example, the prevalence of *T. gondii* was 75.6% in women referred to a prenatal laboratory in Mazandaran [35], 77.4% in AIDS patients [36], and 58.8% in pregnant women [37]. According to a study conducted by Sharif et al. [38] in Mazandaran Province, the toxoplasmosis prevalence in sheep and goats was 35% and 30%, respectively, which is consistent with the high prevalence of *T. gondii* in cats. Geographical location and climatic conditions (including temperature and humidity) are important factors for the survival of oocysts; specifically, this parasite tends to be prevalent in areas with high humidity. The high prevalence of *Toxoplasma* infections in northern Iran may be due to the high humidity of 90% and moderate temperatures of 18-20°C [17].

In conclusion, this is the first study in Iran to provide data related to presence of the Toxoplasmatinae subfamily in dog and cat fecal samples using PCR. The presence of *T. gondii* and *N. caninum* in cat and dog feces in northern Iran indicates a risk of transmission of toxoplasmosis and neosporosis infections. Additionally, the findings of this study suggest that public-health monitoring for the effective control of feces from cats and dogs and improved pet hygiene habits may be needed.

## Figures and Tables

**Table 1.** Summary of primers and annealing temperature for the molecular detection of *N. caninum, H. heydorni, T. gondii, H. hammondi,* and *B. besnoiti* using PCR in this study

Parasite	Primer	Size, bp	Annealing temperature, °C	Time, sec	Reference
*N. caninum*	NP21+: 5’-CCCAGTGCGTCCAATCCTGTAAC-3’	337	63	60	[16]
	NP6+: 5’-CTCGCCAGTCAACCTACGTCTTCT-3’				
*H. heydorni*	JS4: 5’-CGAAATGGGAAGTTTTGTGAAC-3’	267	65	60	[13]
	JS5: 5’-CAGCAGCTAGATACGTAGA-3’				
*T. gondii*	TOX4: 5’-CGCTGCAGGGAGGAAGACGAAAGTTG-3’	529	55	30	[17]
	TOX5: 5’-CGCTGCAGACACAGTGCATCTGGATT-3’				
*H. hammondi*	Hham34F: 5’- ATCCCATTCCGGCTTCAGTCTTTC-3’	282	60	60	[18]
	Hham3R: 5’-ACAGCGGAGCCGAAGTTGGTTTR-3’				
*B. besnoiti*	ITS-1F: 5’-TGACATTTAATAACAATCAACCCTT-3’	230	58	90	[19]
	ITS-1R: 5’-GGTTTGTATTAACCAATCCGTGA-3’				

*N, Neospora; H, Hammondia; T, Toxoplasma; B, Besnoitia*; PCR, polymerase chain reaction.

**Table 2.** Number of positive samples for *T. gondii, N. caninum, Hammondia* spp., and *B. besnoiti*

Animals	*T. gondii*	*N. caninum*	*Hammondia* spp.	*B. besnoiti*
Dog	1/120 (0.8)	2/120 (1.7)	0/120 (0.0)	0/120 (0.0)
Cat	18/80 (22.5)	0/80 (0.0)	0/80 (0.0)	0/80 (0.0)

Values are presented as number (%). *T, Toxoplasma; N, Neospora; B, Besnoitia.*
